# Supplementary material for: Molecular characterization of G-protein-coupled receptor (GPCR) and protein kinase A (PKA) cDNA in Perinereis aibuhitensis and expression during benzo(a)pyrene exposure
Source: PeerJ. 2019 Nov 22;7:e8044. doi: 10.7717/peerj.8044 (PMC6876487; doi:10.7717/peerj.8044)
Supplement: Supplemental Information 1 [file peerj-07-8044-s001.docx]

Real time PCR database

Table1 Real time PCR raw data of GPCR

| day | sample | GPCR | actin | 2 -(∆∆Ct) | average | SD |
| --- | --- | --- | --- | --- | --- | --- |
| 4day | controlO1 | 26.56892014 | 13.24267101 | 0.465697329 | 1.291611206 | 1.087263585 |
|  | controlO2 | 26.15340233 | 14.27365112 | 1.269237834 |  |  |
|  | controlO3 | 25.38464928 | 12.41632175 | 0.596827581 |  |  |
|  | controlO4 | 23.28156662 | 12.56103992 | 2.834682081 |  |  |
|  | acetone1 | 26.03753853 | 12.31532669 | 0.35392162 | 0.883187446 | 0.417800701 |
|  | acetone2 | 25.87490845 | 13.57725143 | 0.95003767 |  |  |
|  | acetone3 | 24.30450821 | 12.53650951 | 1.371462278 |  |  |
|  | acetone1 | 24.51257324 | 12.06677914 | 0.857328216 |  |  |
|  | 0.5BaP1 | 26.37047958 | 12.31484604 | 0.280890569 | 0.880149936 | 0.861765893 |
|  | 0.5BaP2 | 27.37315178 | 12.71744251 | 0.185308938 |  |  |
|  | 0.5BaP3 | 25.78590845 | 13.56714343 | 1.003436013 |  |  |
|  | 0.5BaP4 | 25.7620945 | 14.57468319 | 2.050964226 |  |  |
|  | 5BaP1 | 26.54735756 | 14.93818665 | 1.531072631 | 3.001304003 | 2.458575808 |
|  | 5BaP2 | 25.98607063 | 14.84356785 | 2.115811133 |  |  |
|  | 5BaP3 | 24.90423012 | 13.4356966 | 1.687842306 |  |  |
|  | 5BaP4 | 24.40736771 | 14.9214468 | 6.670489943 |  |  |
|  | 10BaP1 | 23.60482597 | 14.04460144 | 6.335634473 | 4.467824762 | 1.597545744 |
|  | 10BaP2 | 23.95938301 | 13.60657787 | 3.65762837 |  |  |
|  | 10BaP3 | 25.04502678 | 14.2670927 | 2.724099806 |  |  |
|  | 10BaP4 | 24.4701786 | 14.6121397 | 5.153936399 |  |  |
|  | 50BaP1 | 24.49082565 | 14.84082413 | 5.953392904 | 4.072420274 | 1.752348449 |
|  | 50BaP2 | 24.76016998 | 13.97321892 | 2.707127001 |  |  |
|  | 50BaP3 | 24.85181236 | 13.92596149 | 2.458643853 |  |  |
|  | 50BaP4 | 23.8353138 | 13.9819088 | 5.17051734 |  |  |
| 7 days | controlO1 | 23.12096214 | 11.63605785 | 1.668798015 | 1.903355821 | 1.037291433 |
|  | controlO2 | 25.30708694 | 14.69076443 | 3.047003419 |  |  |
|  | controlO3 | 25.45301437 | 14.43143272 | 2.300794268 |  |  |
|  | controlO4 | 25.28564928 | 12.31632175 | 0.598687632 |  |  |
|  | acetone1 | 25.52664948 | 14.38167572 | 2.112190388 | 2.487174497 | 0.573606767 |
|  | acetone2 | 25.53202248 | 14.94830513 | 3.116650304 |  |  |
|  | acetone3 | 24.94538689 | 13.64983654 | 1.902851913 |  |  |
|  | acetone1 | 24.7301178 | 14.00056648 | 2.817005384 |  |  |
|  | 0.5BaP1 | 22.58511734 | 12.09587288 | 3.327570369 | 7.247420389 | 3.680413098 |
|  | 0.5BaP2 | 23.15094376 | 13.33868408 | 5.320102207 |  |  |
|  | 0.5BaP3 | 23.48877335 | 14.3823185 | 8.677375658 |  |  |
|  | 0.5BaP4 | 22.14915848 | 13.46951389 | 11.66463332 |  |  |
|  | 5BaP1 | 24.42656326 | 13.45428085 | 2.380774908 | 6.807582922 | 4.216072815 |
|  | 5BaP2 | 23.02185249 | 13.53380013 | 6.660642125 |  |  |
|  | 5BaP3 | 21.46438789 | 12.88497543 | 12.50385611 |  |  |
|  | 5BaP4 | 23.73571396 | 14.01917553 | 5.685058549 |  |  |
|  | 10BaP1 | 22.71360397 | 13.81892586 | 10.04938647 | 8.391190749 | 1.825384936 |
|  | 10BaP2 | 23.89675331 | 14.35786152 | 6.430013846 |  |  |
|  | 10BaP3 | 23.95235634 | 14.58615017 | 7.247620763 |  |  |
|  | 10BaP4 | 23.84560013 | 14.9202137 | 9.837741914 |  |  |
|  | 50BaP1 | 23.61730385 | 14.21114445 | 7.049662719 | 7.667849796 | 1.919765325 |
|  | 50BaP2 | 24.0920105 | 15.13997459 | 9.657687168 |  |  |
|  | 50BaP3 | 23.9828186 | 14.87699413 | 8.681168024 |  |  |
|  | 50BaP4 | 24.89179802 | 15.06940937 | 5.282881274 |  |  |
| 14 days | controlO1 | 25.86437798 | 14.72588444 | 2.121699146 | 2.031446338 | 0.358894781 |
|  | controlO2 | 25.47229004 | 14.56641006 | 2.492914947 |  |  |
|  | controlO3 | 25.45258522 | 13.97268295 | 1.674594009 |  |  |
|  | controlO4 | 25.51536789 | 14.16867383 | 1.836577251 |  |  |
|  | acetone1 | 25.96198845 | 14.84993649 | 2.16094398 | 2.82416604 | 0.51180338 |
|  | acetone2 | 25.66863251 | 15.17943096 | 3.327669354 |  |  |
|  | acetone3 | 24.71779442 | 13.92939377 | 2.704408312 |  |  |
|  | acetone1 | 24.92731285 | 14.33756161 | 3.103642512 |  |  |
|  | 0.5BaP1 | 22.68990707 | 12.38291359 | 3.775637223 | 9.27370625 | 3.784447986 |
|  | 0.5BaP2 | 21.50157356 | 12.90075302 | 12.31968185 |  |  |
|  | 0.5BaP3 | 22.30780602 | 13.54033184 | 10.97568793 |  |  |
|  | 0.5BaP4 | 22.13489654 | 13.23654312 | 10.02381799 |  |  |
|  | 5BaP1 | 23.74563218 | 14.98564378 | 11.03278598 | 13.13073187 | 2.803298045 |
|  | 5BaP2 | 23.83045387 | 15.69625473 | 17.02419285 |  |  |
|  | 5BaP3 | 24.42510796 | 15.93790436 | 13.32912377 |  |  |
|  | 5BaP4 | 23.34007263 | 14.59362507 | 11.13682489 |  |  |
|  | 10BaP1 | 23.52360344 | 14.23731327 | 7.660419863 | 10.04386968 | 3.516042382 |
|  | 10BaP2 | 23.56570435 | 14.02140999 | 6.405979934 |  |  |
|  | 10BaP3 | 23.73831749 | 15.23744202 | 13.20340534 |  |  |
|  | 10BaP4 | 23.09824181 | 14.56446171 | 12.9056736 |  |  |
|  | 50BaP1 | 23.34560013 | 14.9123137 | 13.83669238 | 13.45736133 | 5.658916745 |
|  | 50BaP2 | 24.67777443 | 15.92118835 | 11.05883542 |  |  |
|  | 50BaP3 | 22.59880829 | 14.77462673 | 21.10528023 |  |  |
|  | 50BaP4 | 21.89512062 | 12.64016819 | 7.828637303 |  |  |

Table2 Real time PCR raw data of PKA

| day | sample | GPCR | actin | 2 -(∆∆Ct) | average | SD |
| --- | --- | --- | --- | --- | --- | --- |
| 4day | controlO1 | 24.14835739 | 17.54047966 | 4.518346276 | 1.619690644 | 0.289244052 |
|  | controlO2 | 23.52828598 | 14.62797165 | 0.922332117 |  |  |
|  | controlO3 | 24.77347755 | 14.46463394 | 0.347438361 |  |  |
|  | controlO4 | 22.79487991 | 13.4772253 | 0.690645823 |  |  |
|  | acetone1 | 25.53218651 | 15.96787357 | 0.582108358 | 0.770509009 | 0.327601708 |
|  | acetone2 | 25.93162155 | 17.4502182 | 1.233082409 |  |  |
|  | acetone3 | 22.8385849 | 13.66947746 | 0.765548208 |  |  |
|  | acetone1 | 24.53000069 | 14.7500658 | 0.501297063 |  |  |
|  | 0.5BaP1 | 26.024365 | 15.9873456 | 0.419473963 | 0.547975605 | 0.346037799 |
|  | 0.5BaP2 | 26.39985085 | 17.53312874 | 0.944060004 |  |  |
|  | 0.5BaP3 | 26.1163826 | 16.53660583 | 0.575902227 |  |  |
|  | 0.5BaP4 | 26.45585442 | 15.68634415 | 0.252466225 |  |  |
|  | 5BaP1 | 22.55252075 | 14.62230778 | 1.806828527 | 2.903033526 | 0.778907529 |
|  | 5BaP2 | 22.86127663 | 15.94275951 | 3.643071875 |  |  |
|  | 5BaP3 | 23.46316719 | 16.33104134 | 3.141702814 |  |  |
|  | 5BaP4 | 22.77191925 | 15.58304882 | 3.020530891 |  |  |
|  | 10BaP1 | 23.31696701 | 14.79746914 | 1.200948845 | 1.953554519 | 0.840871518 |
|  | 10BaP2 | 22.29350471 | 15.15499115 | 3.127823285 |  |  |
|  | 10BaP3 | 23.96678162 | 16.14751244 | 1.951256436 |  |  |
|  | 10BaP4 | 23.79832649 | 15.63213062 | 1.53418951 |  |  |
|  | 50BaP1 | 21.63504028 | 14.26320267 | 2.660753674 | 3.581003845 | 1.172472771 |
|  | 50BaP2 | 21.92193794 | 15.09657764 | 3.886071036 |  |  |
|  | 50BaP3 | 24.04506874 | 16.67547035 | 2.664886674 |  |  |
|  | 50BaP4 | 23.59303093 | 17.16333199 | 5.112303997 |  |  |
| 7 days | controlO1 | 22.26195717 | 13.78885651 | 1.240199239 | 1.66221831 | 0.785211788 |
|  | controlO2 | 23.3449707 | 15.3653717 | 1.746024221 |  |  |
|  | controlO3 | 22.99614525 | 14.11527061 | 0.934844255 |  |  |
|  | controlO4 | 22.99319458 | 15.6572628 | 2.727805525 |  |  |
|  | acetone1 | 23.98365593 | 14.81256008 | 0.764493809 | 1.798828604 | 1.881772293 |
|  | acetone2 | 23.13854027 | 14.34013271 | 0.989838469 |  |  |
|  | acetone3 | 21.53618622 | 14.95973587 | 4.617852919 |  |  |
|  | acetone1 | 23.74234772 | 14.67786598 | 0.823129219 |  |  |
|  | 0.5BaP1 | 24.05022049 | 16.83755302 | 2.971116357 | 2.076486019 | 0.933740724 |
|  | 0.5BaP2 | 24.63015175 | 15.78687096 | 0.959524653 |  |  |
|  | 0.5BaP3 | 25.30461502 | 17.25787354 | 1.666626801 |  |  |
|  | 0.5BaP4 | 23.48639488 | 16.14031029 | 2.708676266 |  |  |
|  | 5BaP1 | 25.53211594 | 17.59922028 | 1.803471858 | 3.158501381 | 2.405485661 |
|  | 5BaP2 | 24.27275467 | 16.96004105 | 2.772060891 |  |  |
|  | 5BaP3 | 23.74136162 | 15.44002628 | 1.39700505 |  |  |
|  | 5BaP4 | 22.84231567 | 16.79448318 | 6.661467725 |  |  |
|  | 10BaP1 | 24.01162338 | 16.21956825 | 1.988413001 | 4.180576092 | 1.91654252 |
|  | 10BaP2 | 24.38273048 | 17.92473984 | 5.013026723 |  |  |
|  | 10BaP3 | 22.84332085 | 16.73239517 | 6.376421049 |  |  |
|  | 10BaP4 | 24.3859005 | 17.34399414 | 3.344443596 |  |  |
|  | 50BaP1 | 24.91988373 | 17.48106194 | 2.540039229 | 4.808544231 | 0.307482729 |
|  | 50BaP2 | 25.7126503 | 17.8923645 | 1.949881938 |  |  |
|  | 50BaP3 | 22.9003067 | 17.7775898 | 12.64903717 |  |  |
|  | 50BaP4 | 23.07778931 | 15.3612175 | 2.095218587 |  |  |
| 14 days | controlO1 | 23.56796265 | 15.12218857 | 1.263914167 | 1.585507792 | 0.621761602 |
|  | controlO2 | 23.74125862 | 15.7868185 | 1.776739846 |  |  |
|  | controlO3 | 22.9888134 | 14.11527061 | 0.939607273 |  |  |
|  | controlO4 | 25.43521118 | 17.89140701 | 2.361769881 |  |  |
|  | acetone1 | 24.40851402 | 15.77689838 | 1.111152577 | 1.393785806 | 0.640836818 |
|  | acetone2 | 24.19967079 | 16.61870193 | 2.301706234 |  |  |
|  | acetone3 | 21.5552578 | 13.19205189 | 1.338360398 |  |  |
|  | acetone1 | 23.82252693 | 14.75943756 | 0.823924016 |  |  |
|  | 0.5BaP1 | 21.43178749 | 14.91952229 | 4.827938725 | 5.052489528 | 1.467868516 |
|  | 0.5BaP2 | 21.72682571 | 15.47898865 | 5.79912608 |  |  |
|  | 0.5BaP3 | 22.91460991 | 15.76336002 | 3.100331936 |  |  |
|  | 0.5BaP4 | 22.95138359 | 16.86427498 | 6.48256137 |  |  |
|  | 5BaP1 | 24.63099098 | 18.79771423 | 7.729611062 | 7.977039266 | 1.606153675 |
|  | 5BaP2 | 24.69756508 | 18.08948135 | 4.517701175 |  |  |
|  | 5BaP3 | 23.04222107 | 18.01273346 | 13.49342758 |  |  |
|  | 5BaP4 | 22.26063919 | 16.10163307 | 6.167417245 |  |  |
|  | 10BaP1 | 23.05692291 | 16.90002632 | 6.17644192 | 4.921655654 | 0.961534612 |
|  | 10BaP2 | 22.21070862 | 15.39868355 | 3.922157584 |  |  |
|  | 10BaP3 | 23.58913574 | 17.15127754 | 5.083472634 |  |  |
|  | 10BaP4 | 23.00327492 | 16.39098549 | 4.504550478 |  |  |
|  | 50BaP1 | 23.47795296 | 17.91802979 | 9.34211626 | 7.961156505 | 2.565201842 |
|  | 50BaP2 | 24.22867012 | 18.20967293 | 6.795950662 |  |  |
|  | 50BaP3 | 21.41007042 | 16.14930725 | 11.49479955 |  |  |
|  | 50BaP4 | 24.63544655 | 17.92619705 | 4.211759549 |  |  |
