# Supplementary material for: Molecular characterization of G-protein-coupled receptor (GPCR) and protein kinase A (PKA) cDNA in Perinereis aibuhitensis and expression during benzo(a)pyrene exposure
Source: PeerJ. 2019 Nov 22;7:e8044. doi: 10.7717/peerj.8044 (PMC6876487; doi:10.7717/peerj.8044)
Supplement: Supplemental Information 3 — The agarose electrophoresis of GPCR RACE product and PKA RACE product [file peerj-07-8044-s003.docx]

PCR database

1. The agarose electrophoresis of GPCR RACE product


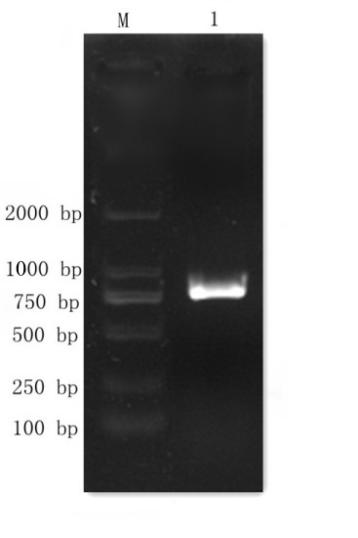

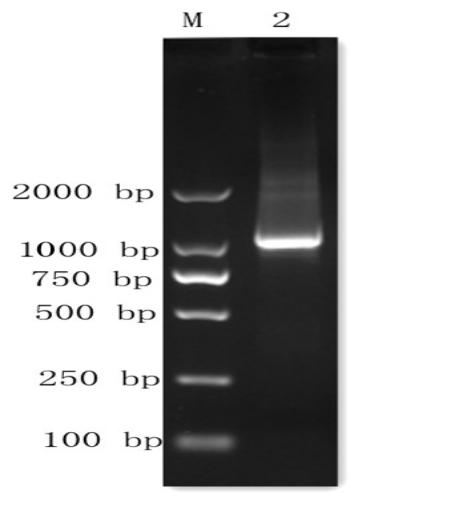


a b

Figure 1 RACE product of GPCR from *P. aibuhitensis*，a shows the result of 3' RACE，b shows the result of 5' Inner RACE

1. The agarose electrophoresis of PKA RACE product


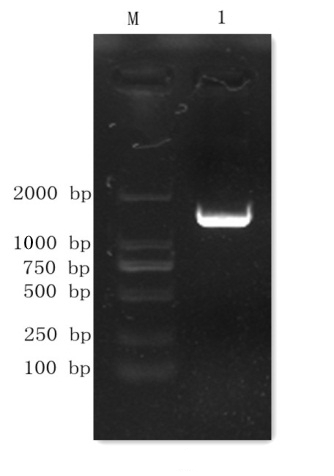

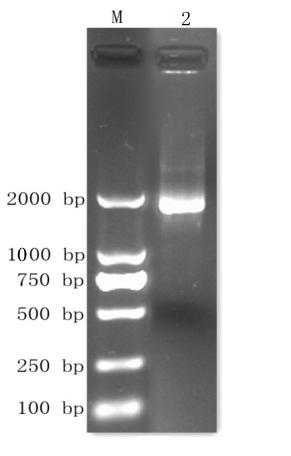


a b

Figure 2 RACE product of PKA from *P. aibuhitensis*，a shows the result of 3' Inner RACE， b shows the result of 5' RACE
