# Supplementary material for: Molecular characterization of G-protein-coupled receptor (GPCR) and protein kinase A (PKA) cDNA in Perinereis aibuhitensis and expression during benzo(a)pyrene exposure
Source: PeerJ. 2019 Nov 22;7:e8044. doi: 10.7717/peerj.8044 (PMC6876487; doi:10.7717/peerj.8044)
Supplement: Supplemental Information 4 [file peerj-07-8044-s004.docx]

# Sequencing Data:

# 1. Name: *Perinereis aibuhitensis* putative gonadotropin-releasing hormone receptor mRNA, complete cds（ *Perinereis aibuhitensis* G-protein-coupled receptors ）

# Accession number: KX792261

# Link for the third part database: <https://www.ncbi.nlm.nih.gov/nuccore/KX792261.1/>

The sequence:

# 1 taaacagtgg cattcacgca gagtacatga gggcagttta agtgattcat tttatgggaa

# 61 tgggtttccc tggtgattag ttagcaaaaa tatatatttc tggaatattt ggacttcaga

# 121 aattgaagga ttttatactt gttcttcggc ttaatggaaa tttcatcttg tgattgagga

# 181 tttaatcggt gaattttatt acatgttaac attatggata acacaacatt caacagaacg

# 241 tttgatggga gtttgaaccc taacttcaac tacattggag attttgtggt gtacatagta

# 301 atcggatgtc ttgggatttt agataatgga tttgttatta tagtcattct ccatagcaga

# 361 aagatgagga ataaactgtg caatttattc atccttaatc aaagtgtggt agacctggtg

# 421 gcttctgtgt ttctcctgtg caattctccg tctgttccga ccttagggtc agcgtcgaat

# 481 attagtctgg agttttattg ccgcatttgg gattcgaact atctcttctg ggctgccgtc

# 541 acatggtcaa cttacaactt agtcgccatc acaatcgaac gttacttaga ggtcgttcac

# 601 ccacttcggt acagatcatt cttcacgcgg agacgtgcaa aggtcattgt cgctgtcgtc

# 661 tggttggttg gattcaccat acctatcgtg acgtcagtta tcaccagtcc tgcgggagca

# 721 gacggcactt gtcagaagca cagagcgtgg tcctcccgac tcatggctgc cctcgtagga

# 781 ttttacgccc tcttcttcgg atttctttta cctgtcgtca taatgatcgt ttgctacact

# 841 cagatgatca tgaccttcaa cttgaaggtc cgaccctccg accccagcac aatgatctcc

# 901 gaaagtgaga aacgtcgaag cgaaaggatg ttgagggtcc gtaaaagcct catcaagaca

# 961 atgttgatgg tttctatcgt ctttgtgatc tgttggatcg gcgaccaagt ttatttcttc

# 1021 ctcttcaaca tcagagtcat caaagacctt caacaaactc ttacgactat cgttgtttcg

# 1081 ttagctttcc tcaattgctg cattaatcca ttcatttaca cctgccaata taacgacttc

# 1141 caagaagcta caagaagact cttaaaaatc aaaaaggaga gtgaaaacag tgaaaggtct

# 1201 acgttggatc tgtctaacca aaaagtctaa tccacatgaa ctaattaaaa catattgatt

# 1261 cgaccaattt ttaactttct tacagattta ccaaaattaa tttttaattt tagttacctc

# 1321 tatttattta atattttcat gtacagcgcc tctgaagatt ttactttccg agatctcgct

# 1381 acataaataa gtcattattt gtatttattt ttaaagaggt aatgacaaat tgtaaagatg

# 1441 ttcttaattt gtgatggtct ccattaaatt tgacatggtc atttactaaa aaaaaaaaaa

# 1501 aaaaaaaaaa aaaa

# Name: *Perinereis aibuhitensis* cAMP-dependent protein kinase A catalytic subunit mRNA, complete cds

# Accession number: KX839259

# Link for the third part database:https://www.ncbi.nlm.nih.gov/nuccore/KX839259

The sequence:

1 gtcgaagaga tcgaggtgga atattcagac ataatttttg agaagctggt tctgagagtt

61 ctctgatttc tggccggtta attcctctgg atcaccacgg actaggtagt ttaccacacg

121 gtagccatgg gaaatgctgc aacagcaaag aaaggcgatc cagagaatgt caaagagttc

181 ttagccaaag ctaaagagga cttcaacaag aaatgggaag agccatcatg taacactgca

241 tcactagatg acttcgacag aattaaaacc ctgggaacag ggtcatttgg acgggtcatg

301 ctggttcagc acaaagccac gaaggagtac tatgccatga agattttaga taaacaaaag

361 gtagtgaaac tgaagcaagt tgaacacaca ttgaatgaaa agaaaattct gtccgccata

421 tcatttccat tcttagtgag cctagagtac agttttaagg ataactcaaa tttgtacatg

481 gtattggagt tcgtgacagg aggtgaaatg ttctcacatc tgcgaagaat tggccgattt

541 agtgaaactc acagccgatt ttatgctgca caagtatgca tggtatttga atatctgcac

601 aatctagaca cactgtacag agatttgaag ccagaaaata ttctgattga tgacactggt

661 cacttgagag taacagactt cggttttgcc aaacgcgtaa aaggcaggac atggacgttg

721 tgtggcacac cagagtacct ggccccagaa atcatcttga gcaagggcta caacaaagcc

781 gtagactggt gggcgcttgg agtccttgtt tatgaaatgg cagctggata cccacctttc

841 tttgctgacc agccaatcca aatctatgag aagattgtct caggaaaggt gcgcttccca

901 tctcacttta gttctgattt gaaggatctt ttgaagaatc tgctacaggt agacttgaca

961 aaacgttatg gaaacctgaa gaatggggtc aacgatatca agaatcacaa gtggttctcc

1021 accacagact ggattgctat ctaccagaaa aaggttgaag cacccttcat tcccaagtgc

1081 aaaggcccag gtgactacag caactttgat gactatgagg aagaaccact gagaatttcg

1141 tcaacggaaa aatgtgccaa ggagtttgca gacttctgag acagttggct tgtaggtggc

1201 tagtgtgtgt tgggggaact aaagactgtg atgaccattc tgtgttcatg gtctatcact

1261 ggcagctggc tgaagatctg cggcccacat accacacaat ctctcaaaga atacatcctt

1321 tgtgcattaa caaatgtata tatacttaat catatcacat tgcgtaattg taacaaggtg

1381 aattcagttt catatgtgtc aatgaggtaa gagagagggc ttcgatccca ggtgtggcat

1441 gtccctgagg ctgcgctatt ggtccgaggt aagccaggtc gcttcagctc gcaccccagg

1501 ctgcctcaaa agctaccttg gtcgtctact cgccgaacta gtcatttacc ttgctgtgtg

1561 gccttccaga tttgtgtggg gcagactggt ggaggattag gctttaattg ctttgctgaa

1621 gtgccccaca cgggtccaga agccaaggct atcttcaaat acacagttaa taccatatat

1681 taacttatca ttacaacctt actgaccact tgttcacatt taggtcattg tctgcacttt

1741 tattgtatac aggtggtcca tctgaaacaa ttctctgaac atttgaatta tatccattgt

1801 aaggaagtgc aaacttctct tgtgctgtgc tcctctccac tcccgtgctg cttctttctc

1861 tgctgtgaac tttgaatcag agagaaacaa cactttattt gtatgatgtc aaatattgta

1921 cttttggtaa ggccacctcc taactgggtg gtcatcaaga cttgtatata gtttgtgagc

1981 agttggcttt tgggtagagt aggagttttc tgttcatcat actggtggga tgacaccatc

2041 tcccgcatac cagtagtact gtgtagtagc aatagccggc cataaggcct atgagtctcc

2101 attgtttcag ctaatgtagc taggcttcaa gtcataagtc atgataacat ataatggcat

2161 tatttgtagt actgatgatg agagaatatt atctttgtca agacaaactt attttacttt

2221 tatataactg acttgtatgt acagacgtca aggctgtatg aactgagaaa ttgttgcttg

2281 ttatgtgtag tgtataaaat gtgtatatct atctctgcat attttgtgtt tgattgtcaa

2341 tccacaaatg cttcaaattt atcttgaaac catgtacttg actaatctaa tttgtgattt

2401 gaaaaggtaa ttggagttgt gctgaattag ataggtatgc ttcctgacat tgtgtaacac

2461 tactacagtg agtcccattt gtcatgggtc ctactcatga atgttgttat attttgtata

2521 tactaaaaat aatttatgct ggttcatgaa gatgtacaaa aaaatatttt tcaaatggct

2581 aaggatggtt ttgatgaaat tgttacacta agggtcagca gttaacaaac cccatgaaaa

2641 aaaaaaaaaa aaaaaaaaaa aa
